# Perforated Meckel's Diverticulum Lithiasis: An Unusual Cause of Peritonitis

**DOI:** 10.1155/2013/825628

**Published:** 2013-05-29

**Authors:** Umasankar Mathuram Thiyagarajan, Amirthavarshini Ponnuswamy, Atul Bagul, Prabakar Ponnuswamy

**Affiliations:** ^1^Department of General and Transplant Surgery, Derriford Hospital, Plymouth PL6 8DH, UK; ^2^Royal Cornwall Hospital, Truro TR1 3LJ, UK; ^3^St George Hospital, London SW17 0QT, UK; ^4^Sri Laxmi Hospital, Tirupattur 635601, India

## Abstract

Meckel's diverticulum is the commonest congenital malformation of gastrointestinal tract and represents a persistent remnant of the omphalomesenteric duct. Although it mostly remains silent, it can present as bleeding, perforation, intestinal obstruction, intussusception, and tumours. These complications, especially bleeding, tend to be more common in the paediatric group and intestinal obstruction in adults. Stone formation (lithiasis) in Meckel's diverticulum is rare. We report a case of Meckel's diverticulum lithiasis which presented as an acute abdomen in an otherwise healthy individual. The patient underwent an exploratory laparotomy which revealed a perforated Meckel's diverticulum with lithiasis; a segmental resection with end-to-end anastomosis of small bowel was performed. Patient recovery was delayed due to pneumonia, discharged on day 20 with no further complications at 6 months following surgery.

## 1. Introduction

German anatomist Johann Friedrich Meckel first described the embryological and pathological features in 1809 [1]. The incidence of Meckel's diverticulum (MD) varies between 1 and 2% and carries the lifetime risk of 4–6% to become symptomatic. In the case described, the patient was initially managed conservatively by the general practitioner (GP) as presenting symptoms were vague, but as the patient dramatically deteriorated within the next 24 hours a surgical opinion was sought. This case thus reminds the need for an open mind, centred on a differential diagnosis without forgetting rare diseases entities like Meckel's diverticulum and its associated complications.

## 2. Case Presentation

A 78-year-old man was admitted with a right-sided abdominal pain of 2 days duration followed by nausea and vomiting with fever. He had seen his GP with low grade pain in the right lower abdomen previously in the week and treated conservatively as blood investigations were within normal range. Before this illness, the patient was a healthy individual with no previous medical history. On the day of admission he was febrile (37.9°C) with hypotension (100/65) and tachycardia (95/minute). Abdominal examination showed mild distension, tenderness, and guarding in the right lower quadrant, with tinkling bowel sounds. The digital rectal examination and urine dipstick tests were normal. 

Clinical examination of other systems to include cardiovascular, respiratory, genitourinary, and central nervous systems was unremarkable. 

## 3. Investigations

The blood investigations were within normal limits and including a full blood count, inflammatory markers, and renal and liver function tests. Plain abdominal and chest X-ray films did not reveal any abnormalities; ultrasonography of abdomen showed some free fluid in the pelvis. 

Abdominal computerized tomographic (ACT) scan confirmed the presence of minimal amount of intraperitoneal free fluid, nondilated small bowel loops, and absence of free gas in the peritoneal cavity. There was a calcified nodule in the right lower quadrant which was reported as a possible calcified mesenteric lymph node. Some inflammatory changes in the distal small bowel wall were noted, and the appendix was visualized as normal. The patient was thus managed conservatively with broad spectrum antibiotics and serial abdominal examination.

## 4. Treatment

The patient received intravenous fluids, antibiotics (Co-Amoxiclav 1.2 gram 8th hourly intravenously), and pain relief as planned conservative regimen. After 12 hour, the patient failed to improve and continued to be febrile, and sequential abdominal examination showed features of localized peritonitis. 

**Figure 1:**
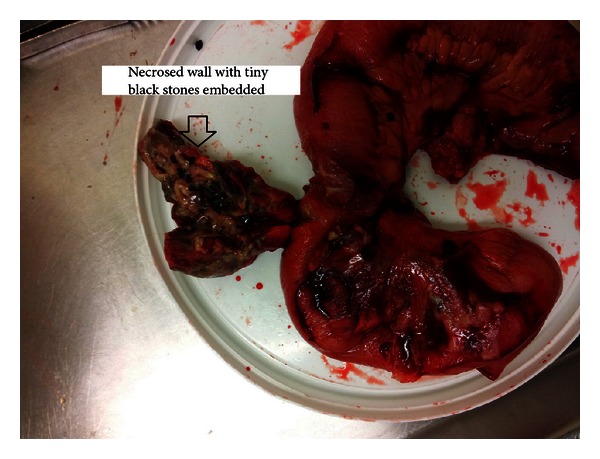
Necrosed Meckel's diverticulum with multiple stones.

A diagnostic laparoscopy (DL) was thus performed, which showed an inflammatory mass in the right iliac fossa. The DL was then converted to lower midline laparotomy. A Meckel's diverticulum was found approximately 50 centimetres from ileocaecal valve; half the circumference of the wall of Meckel's diverticulum was necrosed (see Figure 1). There was a small collection in the peritoneal cavity which contained clusters of multiple black coloured stones. Some of the stones were well embedded with the wall of the diverticulum and stones weighed 39 grams in total. Segmental resection of this affected segment of small bowel with end-to-end anastomosis was thus carried out. 

Histopathological examination confirmed the presence of diverticulitis and extensive mucosal necrosis possibly due to ischaemia in the wall without heterotopic mucosa. But no abnormality was found in small- or medium-sized blood vessels. Unfortunately a biochemical analysis of the stones was not performed.

The patient had a delayed recovery due to pneumonia and was discharged on day 20 with no further complications at 6 months following surgery.

## 5. Discussion

Symptomatic lithiasis in the Meckel's diverticulum is very rare [1, 2]. Park et al. reported that among adult patients, the most common presentations of symptomatic Meckel diverticula were bleeding, obstruction, and diverticulitis. Diverticulitis was present in 28% patients; this included 10% who presented with perforated viscus and only 1% involved perforations secondary to a foreign body [3]. Kusumoto et al.'s review of 776 patients with Meckel's diverticulum showed two cases to be associated with stones [4].

The pathogenesis of lithiasis in Meckel's diverticulum remains unclear. It may be related to stasis resulting from poor coordination of the peristaltic wave at the site of Meckel's diverticulum [5, 6]. The absence of ectopic gastric mucosa may also lead to a more alkaline environment in the diverticulum, favouring precipitation of calcium and other minerals necessary for lithiasis [7, 8]. The “stone” itself may usually be either a fecalith, bezoar, or gallstone.

Diagnosing lithiasis or associated complication from lithiasis within a Meckel's diverticulum radiologically can be difficult, and this needs to be considered within the differential diagnosis of abdominal calcifications [2]. Abdominal CT scans in current day may be helpful to diagnose a Meckel diverticulum but may overlook associated lithiasis because of the lack of typical radiological features as was the case in the present report. Our patient had necrotizing Meckel's diverticulum with localized abscess and mass formation.

Lithiasis of Meckel's diverticulum is a rare complication. Nevertheless, this entity should be included in the differential diagnosis of abdominal calcification when a peripheral calcified lesion is detected in the lower abdomen on ACT scan of adults with acute abdomen. 

Pathogeny, how multiple stones somehow led to necrosis and perforation, remains unclear. Inflammation and necrosis in a Meckel's diverticula secondary to a stone have been reported [9]. This is usually due to a single large stone causing inflammation and pressure necrosis. Although stone analysis was not carried out in the case reported, the stones may be fecaliths, bezoars, or gallstones. Likely scenarios are that the Meckel's diverticulum perforated secondary to inflammation and ischemia; these “stones” were an incidental finding or they could be related to a mass effect from multiple stones.

## 6. Learning Points and Take Home Messages

- Meckel's diverticulum lithiasis can present as an atypical cause of peritonitis.
- Abdominal computerised tomography scans may be useful to diagnose a Meckel's diverticulitis but may not add helpful information with its associated complications such as lithiasis.
- Diagnostic laparoscopy has a role in these atypical presentations in diagnosis and definitive treatment.
